# Spinal hydatid cyst initially diagnosed as spinal tumor: A case report and review of the literature

**DOI:** 10.1002/ccr3.7244

**Published:** 2023-05-01

**Authors:** Alireza Zali, Mohammadreza Shahmohammadi, Bijan Herfedoust Biazar, Niloofar Masoumi, Noosha Samieefar, Meisam Akhlaghdoust

**Affiliations:** ^1^ Functional Neurosurgery Research Center, Shohada Tajrish Neurosurgical Comprehensive Center of Excellence Shahid Beheshti University of Medical Sciences Tehran Iran; ^2^ USERN Office, Functional Neurosurgery Research Center Shahid Beheshti University of Medical Sciences Tehran Iran; ^3^ Student Research Committee, School of Pharmacy Shahid Beheshti University of Medical Sciences Tehran Iran; ^4^ USERN Office Shahid Beheshti University of Medical Sciences Tehran Iran; ^5^ Network of Interdisciplinarity in Neonates and Infants (NINI) Universal Scientific Education and Research Network (USERN) Tehran Iran; ^6^ School of Medicine Shahid Beheshti University of Medical Sciences Tehran Iran

**Keywords:** case report, hydatid cyst, hydatidosis, parasitic infection, spinal hydatidosis

## Abstract

The spinal hydatid cyst is a benign pathology but has considerable morbidity. It should be considered as a differential diagnosis in patients having signs and symptoms of spinal compression, particularly in endemic areas.

## Abstract 2

Hydatid cyst is a parasitic infection caused by larvae of Echinococcus spp. Approximately 60%–70% of patients with spinal hydatidosis have lesions in the liver, 15%–20% have lung involvement, and only 0.5%–2% have bone involvement. Primary spinal hydatid cysts account for about 1% of cases. A 38‐year‐old woman with a history of back and chest pain that eventually led to numbness in the abdomen and legs was admitted to Iranmehr Hospital. The patient had a positive family history of hydatid cysts, and her brother had two pulmonary infestations when he was 40 years old. The cyst was not recognizable with MRI, and the patient was first admitted to diagnose a spinal cord tumor. During the surgery, a hydatid cyst was recognized. The cystic lesion was completely removed without any cystic rupture. Spinal hydatidosis is a rare disease with considerable morbidity and poor prognosis. It should be considered as one of the differential diagnoses when approaching a patient with spinal compression syndrome.

## 1 INTRODUCTION

Hydatid cyst is a parasitic infectious disease caused by larvae of *Echinococcus granulosus cestode*.^1^ This infection is more prevalent in areas where livestock and agriculture are common. Mortality from this cyst ranges from 0.9% to 3.6%.^2^ It can infest all organs and tissues; almost 60%–70% of the hydatid cysts are formed in the liver, while about 15%–20% involve the lungs. Bone involvement is rare and beholds only in around 0.5%–2% of cases, nearly half of which are located in the vertebrae.^1^, ^3^ Spinal cord involvement is rare and has a poor prognosis. Mostly, the thoracic vertebra is involved.^4^

Clinical symptoms are usually latent and not specific and correlate with the location and size of the lesion. The symptoms may appear when the lesions become large. The symptoms range from radicular pain to bone fractures.^2^, ^5^ Generally, we have five locations of spinal hydatid cases described as intramedullary, intradural extramedullary, extradural, vertebral, and paravertebral lesions.^6^, ^7^ Surgery is the prior treatment for spinal echinococcosis. Provided that a patient is suspected of this infection, biopsy or aspiration should be avoided due to the diffusion and anaphylaxis risk.^2^ This study presents a patient with a spinal hydatid cyst, who was first admitted with a suspicion of a spinal tumor. A narrative review of spinal hydatidosis cases will also be provided.

## 2 CASE PRESENTATION

A 38‐year‐old Iranian woman with a history of back and chest pain that eventually led to numbness in the abdomen and legs was admitted to Iranmehr Hospital in December 2021. Symptoms developed in 6 months. The patient did not have a pet and had no history of underlying diseases. Her last pregnancy was 7 years ago. The patient had a history of axillary surgery. Her family history revealed that the patient's brother had thoracic hydatid cysts at the age of 40, both of which were pulmonary cysts in different lungs.

**FIGURE 1:**
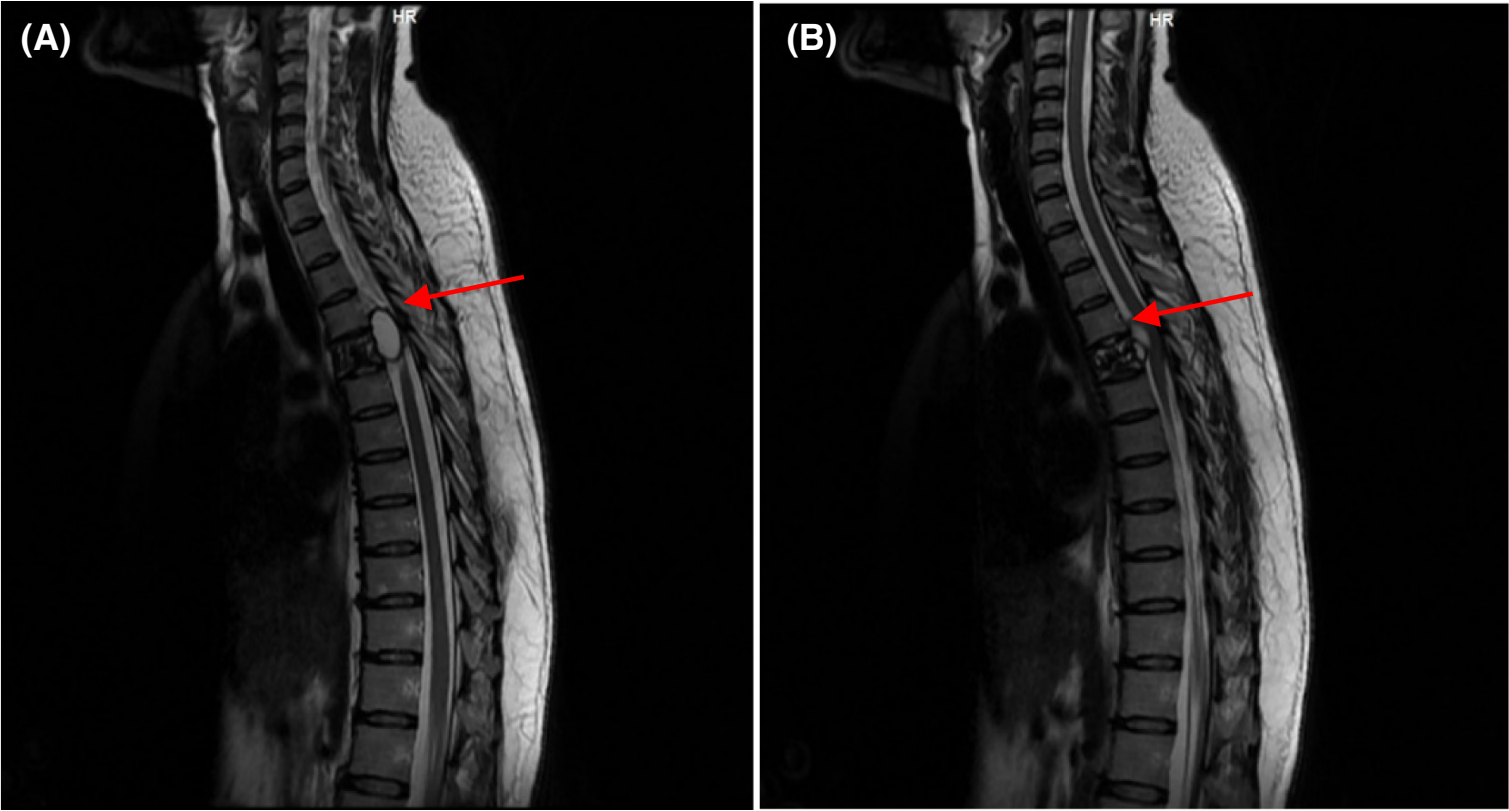
Magnetic resonance imaging (MRI) of the thoracic spine: (A) Hydatid cyst and (B) vertebral fracture.

The primary diagnosis was a spinal cord tumor at the T5 level, most likely an epidermoid one, since the cyst was not recognized to be echinococcosis with MRI primarily. Components of the cyst in sagittal cross‐sections of T5 had caused damage and fracture of the thoracic vertebrae in this area (see Figure 1).

During the surgery, it turned out that the lesion was a hydatid cyst. Thereafter, pathologic investigations confirmed the diagnosis. The report was from an extra‐medullary intradural specimen. The microscopic finding was acellular lamellated and germinative layers accompanied by some scolices.

The patient underwent surgery, and the cystic lesion was completely removed without any rupture via hemilaminectomy of the T5 vertebrae from the left side. In addition, two‐stage combined anterior and posterior decompression was performed. The surgical region was washed off using 20% hypertonic saline during the same session. The patient received albendazole 10–15 mg/kg/day postoperatively for 6 months. In 6 months of follow‐up, the patient had no related complaints, and her back and chest pain resolved.

## 3 DISCUSSION

Echinococcosis is a zoonotic and chronic infection; it occurs worldwide, usually in tropical and subtropical regions. Therefore, it is difficult to determine the exact prevalence and number of infected patients.^2^, ^8^ Hydatid infection mainly forms in the liver and lungs, as these organs trap most of the larvae. Spinal hydatid cyst is rare and occurs in fewer than 1% of patients with hydatidosis. In spinal cord involvement, clinical manifestations depend on the level of vertebrae involvement and the stage of the disease.^9^, ^10^ The spinal echinococcosis symptoms depend on how much compression applies to the spinal cord. Some nonspecific symptoms include back pain, sensory discomfort, dysuria, etc. Previous studies have demonstrated that patients also suffer from paraplegia.^2^, ^11^ Consequently, when a bone is fractured, neurological deficits and pain arise together.^1^ A summary of recent studies on spinal hydatid cysts is available in Table 1.

**TABLE 1:** Summary of studies on spinal cord hydatid cyst.

Authors	Year	Country	Age	Sex	Clinical manifestations	Radiological investigations	Operative findings	Consequence
Çeviker et al.^13^	2022	Turkey	63	Male	Lost bilateral lower extremities strength, paraplegia, immobilization, and swelling in the thoracolumbar region	In MRI thought to be metastasis, involving the dorsal colon, right pedicle and lamina, and soft tissue densities. Abdominal CT showed a hypodense cystic mass lesion with necrotic, hyperdense calcific foci extending towards the spinal canal at the T 11 level	Hyperdense calcific foci the spinal canal in the T11	Improved
Santos et al.^14^	2022	Brazil	22	Male	Paresthesia, numbness in the chest, lower limb paraparesis, and back pain in a sitting position	CT and MRI showed multiloculated cysts extending to the entire length of the second right costal arch, especially to the intrathoracic. The lytic lesion was found on the body and posterior arch elements of the T2 vertebra and the T1 and T3 posterior elements	Paraspinal soft tissue and intracanal extradural lesions were extending T1/T2 and T3/T4 causing stenosis and compression of the spinal cord with edema	Improved
Kalyanasundarabharathi et al.^15^	2021	India	64	Female	Painless swelling in the mid‐back since childhood, sudden inability to walk for 1 week started, weakness of both lower limbs, inability to pass urine, and constipation for 1 week	Ultrasound examination of the abdomen and pelvis and CT of the brain and chest were regular	Intraoperatively, a hydatid cyst sized 15 × 10 cm in the left paraspinal muscles was found (erector spinae) with intraspinal extradural extension into D8–D12 compressing the dorsal cord D8–D9 as an osteolytic lesion extending into the spinal canal	Improved
Safari et al.^9^	2021	Iran	38	Male	Back pain, chest pain, paraplegia, and urinary incontinence		– Surgical removal was performed with the costotransverse approach, and multiple epidural cystic lesions at the T3–T4 level were completely resected	– Improved
						CT showed multiple cysts located behind the left lung and the fourth rib		
						MRI revealed multiple spinal epidural cystic lesions at the level of the third to fourth thoracic vertebrae		
Das et al.^3^	2021	India	35	Male	Back pain and intermittent fever	Kyphosis in the D2–D6 and partial collapse of D4 were found in the X‐ray result. hydatid cyst disease was confirmed in the D4 vertebra involving the paravertebral area	In the paravertebral area, intact cysts were excised	Improved
Saul et al.^16^	2020	Syria	42	Male	Middle thoracic and paravertebral pain	Lytic progression in T8 with high‐grade constriction of the spinal cord has been showing in MRI	The T8 vertebral body was interfused by white granular tissue	Improved
Mansfield et al.^17^	2019	South Africa	38	Male	Chronic lower back pain radiating down the back of his legs	Vertebral body destruction of the L4 vertebra with multiple surrounding cysts was shown in MRI	N/A	Improved
Saha et al.^18^	2018	India	22	Male	Progressive backache weakness of both lower limbs	Mixed hypodense and hyperdense epidural soft tissue mass lesions with few fluid intensities were found in MRI	Extradural intraspinal lesion	Improved
Kandwal et al.^19^	2018	India	30	Male	Low back pain for 3 years with radicular pain in the left lower limb and a history of severe allergic skin rashes over the body	Bone destruction was eccentric with an absence of L3 pedicle shadow on the left side in the anteroposterior was viewed in MRI. The bone involvement was situated up to 50% in the lateral view of the radiograph. and an Osteolytic lesion with an absence of L3 pedicle on the left side was observed in the CT scan	The lesion was extradural and mostly confined to the L3 vertebra	Improved
Unal et al.^20^	2017	Turkey	43	Female	Fluid exudation from a cutaneous fistula on the left hip and left leg pain	A heterogeneous low‐signal intensity sacral lesion was observed on MRI Lytic lesions in bilateral wings of the S1 vertebra in CT scan	The lesion location was in the vertebral body at the S1 level and extended to the foramen and paravertebral muscle	Improved
Xia et al.^21^	2009–2016	China	47 50 43 32 69	Female Male Male Female Male	Backpain Backpain Headache N/A Abdominal pain	Lesion location was in T7–T9 Lesion location was in L3–L5 The lesions were found in the brain and T11‐L5 Spinal cord compression at T3‐S5 level Lesions located in the spinal canal at the L3–L5 level	– – – – –	Improved Paresis Death Death Improved
Meinel et al.^22^	2016 Published in 2017	Switzerland	75	Female	Atypical low back pain and substantial weight loss	CT scan showed osteolytic lesions in the lumbar vertebrae L3 and L4 was founded in CT scan, and a multilobulated osteolytic cystic mass was observed in the lumbar and paravertebral region	Extradural granulation and purulent fluid at L1–L4 with the demolition of laminae	Improved
Vizcarra et al.^23^	2016	Peru	14	Male	Progressive hypoesthesia	Destruction and compression of L5 to S2 were found in MRI and Multilocular cystic tumor of L5 to S2 vertebrae in CT scan	Multiple thin‐walled hydatid cysts infiltrated the bone and dural sac (L5‐S2)	Improved
Abbasi et al.^24^	2016	Iran	61	Male	Intense and continuing low back pain	The MRI results showed high signal intensity, that is, the expansile lesion in T2‐weighted images and low signal intensity in T1‐weighted images. Moreover, expansile lytic lesions in the sacral bone were observed	Uniloculated lytic lesion with minimal soft tissue extension	Improved
Gennari et al.^25^	2016	South of Romania	25	Female	Backache and slight weakness of both legs, paravertebral tumefaction	Costovertebral destruction of the T9 vertebra was observed in the CT scan. Multiple cystic lesions were found that invade the spinal canal producing spinal cord compression at level T9 in MRI	Complete extradural cyst	Improved

In this case report, our patient had back pain that led to numbness in the abdomen and legs. CT scan and MRI work‐ups confirmed a spinal lesion. Although the patient was admitted with spinal tumor diagnosis, other possible differentials were also considered. Highlighting that the patient's family history of infestation was positive, biopsy was not performed. Therefore, the hydatid cyst was not diagnosed till surgery. MRI is the modality of choice in the radiologic examination. However, when the typical appearance is not present, it would lead to misdiagnosis.^12^

The gold standard treatment for hydatid cysts is surgery, by eliminating the whole cyst. Therefore, timely and accurate diagnosis results in choosing the best treatment. Even if the patient is doubtful of spinal echinococcosis, biopsy and aspiration of the cyst are not recommended because of diffusion risk and anaphylaxis that might happen.^2^, ^9^ Various solutions are used for washing the surgical region, and postoperative adjuvant antiparasitic chemotherapy consists of hypertonic saline (3%, 10%, 20%), 0.5% betadine, 0.5% silver nitrate, and 2% formalin.^1^, ^10^ Albendazole pharmacotherapy is recommended after surgery for 6 months to 1 year to prevent any recurrence of the cyst. For our patient, albendazole was prescribed for 6 months under the supervision of an infectious disease specialist.

## 4 CONCLUSIONS

The spinal hydatid cyst is a benign pathology but has considerable morbidity. It should be considered as a differential diagnosis in patients having signs and symptoms of spinal compression, particularly in endemic areas. Furthermore, correct and timely preoperative diagnosis and suitable surgical techniques according to the cyst features are essential in preventing recurrence.

## AUTHOR CONTRIBUTIONS

**Alireza Zali:** Conceptualization; methodology; supervision; writing – review and editing. **Mohammadreza Shahmohammadi:** Conceptualization; methodology; writing – review and editing. **Bijan Herfedoust Biazar:** Conceptualization; writing – review and editing. **Niloofar Masoumi:** Conceptualization; writing – original draft. **Noosha Samieefar:** Methodology; writing – original draft; writing – review and editing. **Meisam Akhlaghdoust:** Conceptualization; methodology; supervision; writing – review and editing.

## CONFLICT OF INTEREST STATEMENT

The authors declare that they have no conflict of interest.

## CONSENT

Written informed consent was obtained from the patient to publish this report in accordance with the journal's patient consent policy.

## Data Availability

All data of our present case are available in the manuscript.
